# Transitional lumbosacral vertebrae in black Norwegian elkhound and Brittany dogs: Clinical findings and its association with degenerative lumbosacral stenosis

**DOI:** 10.1186/s13028-025-00797-7

**Published:** 2025-02-12

**Authors:** Jon Andre Berg, Bente Kristin Saevik, Frode Lingaas, Cathrine Trangerud

**Affiliations:** 1 Department of Preclinical Sciences and Pathology, Faculty of Veterinary Medicine, Norwegian University of Life Sciences, Oluf Thesens Vei 30, Ås, 1432 Norway; 2 AniCura Jeløy Dyresykehus, Varnaveien 43d, Moss, 1526 Norway; 3 Department of Companion Animal Clinical Sciences, Faculty of Veterinary Medicine, Norwegian University of Life Sciences, Oluf Thesens Vei 30, Ås, Norway; 4 Veterinaerradiologene AS, Skytta terrasse 2, Hagan, 1481 Norway

**Keywords:** Canine, Cauda equina syndrome, Computed tomography, CT, Diagnostic imaging, Magnetic resonance imaging, MRI

## Abstract

**Background:**

Lumbosacral transitional vertebra (LTV) is a congenital anomaly in dogs and have been proposed to be associated with cauda equina syndrome (CES) in German Shepherd dogs. This prospective study, including 32 dogs from two breeds, aims to investigate if LTV is associated with lower back pain in dogs. The study compared owners’ assessment of their dogs’ well-being and clinical evaluation with advanced diagnostic imaging to identify changes that might explain clinical findings.

**Results:**

Dogs with LTV type 2 (57.0%) and with LTV type 3 (70.0%) exhibited mild lower back pain, which was significantly more frequent (*P* = 0.012) compared to dogs with LTV type 0 and LTV type 1. Advanced diagnostic imaging identified a plausible cause for this pain. Dogs diagnosed with LTV types 2 and 3 with lower back pain tended to be lighter (median 14.50 kg) and younger (median 4.10 years) than breeds typically reported for degenerative lumbosacral stenosis (DLSS). Based on the owners’ assessment of their dogs, they considered them pain-free.

**Conclusions:**

The study identified a correlation between LTV types 2 and 3 and lower back pain in these dog breeds. Advanced diagnostic imaging findings confirmed that dogs with these LTV types were more likely to exhibit pathological changes associated with DLSS.

**Supplementary Information:**

The online version contains supplementary material available at 10.1186/s13028-025-00797-7.

## Background

Lumbosacral transitional vertebra (LTV) represents a congenital anomaly [1], with reported occurrences varying between breeds and studies, ranging from 2.3% to 66.86%, although most breeds have an occurrence between 4.0% and 12.0% [2–7]. There are several classification systems for LTV in dogs [3, 4, 8]. A classification based on ventrodorsal (VD) radiographs has been developed, where LTV Type 1 is characterised by an independent spinous process of the first sacral vertebra, which is separated from the medial sacral crest. Type 2 is a symmetrical form in which the transverse processes are partially fused with the sacrum or ilium, but the vertebral body is separated from the sacrum. Type 3 is asymmetrical, where one side typically resembles a normal lumbar vertebra, while the contralateral side resembles the sacral wing, which typically articulates with the ilium. Lastly, LTV Type 0 indicates normal lumbosacral anatomy [8].

Cauda equina syndrome (CES) is a collection of neurological signs resulting from a primary or secondary lesion affecting the cauda equina itself or the peripheral nerves originating from it. It may also arise from primary or secondary disease affecting the lumbar vertebrae, sacral vertebrae, caudal vertebrae, or surrounding soft tissues. Consequently, this syndrome can be categorised as either primary or secondary, depending on its relationship to the spinal cord and nerve roots [9, 10].

Causes of CES may include neoplasia, discospondylitis, epidural empyema, tethered cord syndrome, epidural lipomatosis, and epidural or para-synovial cysts, in addition to degenerative lumbosacral stenosis (DLSS) [11–15]. Degenerative lumbosacral stenosis is a multifactorial degenerative disorder resulting in stenosis of the spinal canal and compression of the cauda equina or its blood supply and is the most common cause of CES [16–18].

Clinical signs related to DLSS are often vague and heterogenous depending on severity. Lumbosacral pain, hyperesthesia in the lumbosacral region, difficulty with rising, jumping, or entering a car, and unilateral or bilateral pelvic limb lameness, which may sometimes be non-weight-bearing are reported. Posterior paresis is also observed, while a positive lordosis test is frequently the most consistent finding [16, 19, 20]. Profound neurological deficits are uncommon but, when present, typically include lower motor neuron signs in the pelvic limbs [21]. Urinary and faecal incontinence may occur in severely affected dogs [22]. A positive lordosis test is sensitive, but not specific to DLSS [20, 23, 24].

The diagnosis of DLSS is based on a combination of history, clinical signs and advanced diagnostic imaging findings after the exclusion of related differential diagnoses [25–27]. Findings on advanced diagnostic imaging modalities considered as part of DLSS have been described [28–34]. It should be noted that there is no significant correlation between the severity of clinical signs and findings seen on magnetic resonance imaging (MRI), and there is only moderate agreement between surgical findings and findings on MRI and computed tomography (CT) [19, 35, 36]. Currently, there is no consensus on the criteria for an advanced imaging diagnosis of DLSS, and the validation of imaging findings is hindered by the absence of a clearly defined disease definition [18].

Heavy work and training have been reported as risk factors [17, 20, 23], and DLSS is particularly reported among middle-aged and old dogs of medium to large dog breeds [18, 23, 37].

Degenerative lumbosacral stenosis is a relatively common reason for veterinary visits often causing chronic pain, leading to reduced quality of life, altered behaviour and leading to early retirement from active duty among military and police working dogs [38–41].

The lumbosacral (LS) area experiences dynamic motion and is subjected to repeated compression and torque during canine locomotion [42]. LTV may alter this motion and has been reported to contribute to developing CES in German Shepherd dogs (GSD) [43, 44].

Another potential cause of pain in the LS area is pathology related to the sacroiliac joint and its soft tissue structures [45–49], where pathology related to the sacroiliac joint based on CT and MRI has recently been described [49–51]. There are inconsistencies related to the possible effect of asymmetric LTV and pathology related to the sacroiliac joint in dogs [33, 43, 48].

In humans, the Castellvi classification categorises LTV based on the morphology of the transverse processes and their relationship to the sacrum and / or ilium. This classification is divided into four types, each further subtyped as “a” or “b” based on whether the anomaly is unilateral or bilateral [52]. Castellvi type 2 LTV exhibits either incomplete unilateral (2a) or bilateral (2b) lumbarisation or sacralisation. This involves an enlarged transverse process with a diarthrodial joint between it and the sacral wing, which is often associated with lower back pain [53]. In rarer cases, excessive bone formation between the LTV body and the sacral wing can lead to nerve root entrapment, referred to as “extraforaminal nerve entrapment” [54]. This condition may resemble “far-out syndrome,” where osteophytes at the articulation between the transverse process of the transitional vertebra and the sacrum cause entrapment of the L5 nerve root [55].

Limited research exists on potential links between LTV and lower back pain in dogs. In Norway, this lack of information has raised health concerns among dog owners and breeders about the impact of LTV.

In this prospective study, we hypothesised that LTV type 2 and LTV type 3 in Norwegian Elkhound black and Brittany could be associated with clinical signs of lower back pain, supported by findings identified through advanced diagnostic imaging.

## Methods

### Dogs

**Fig. 1:**
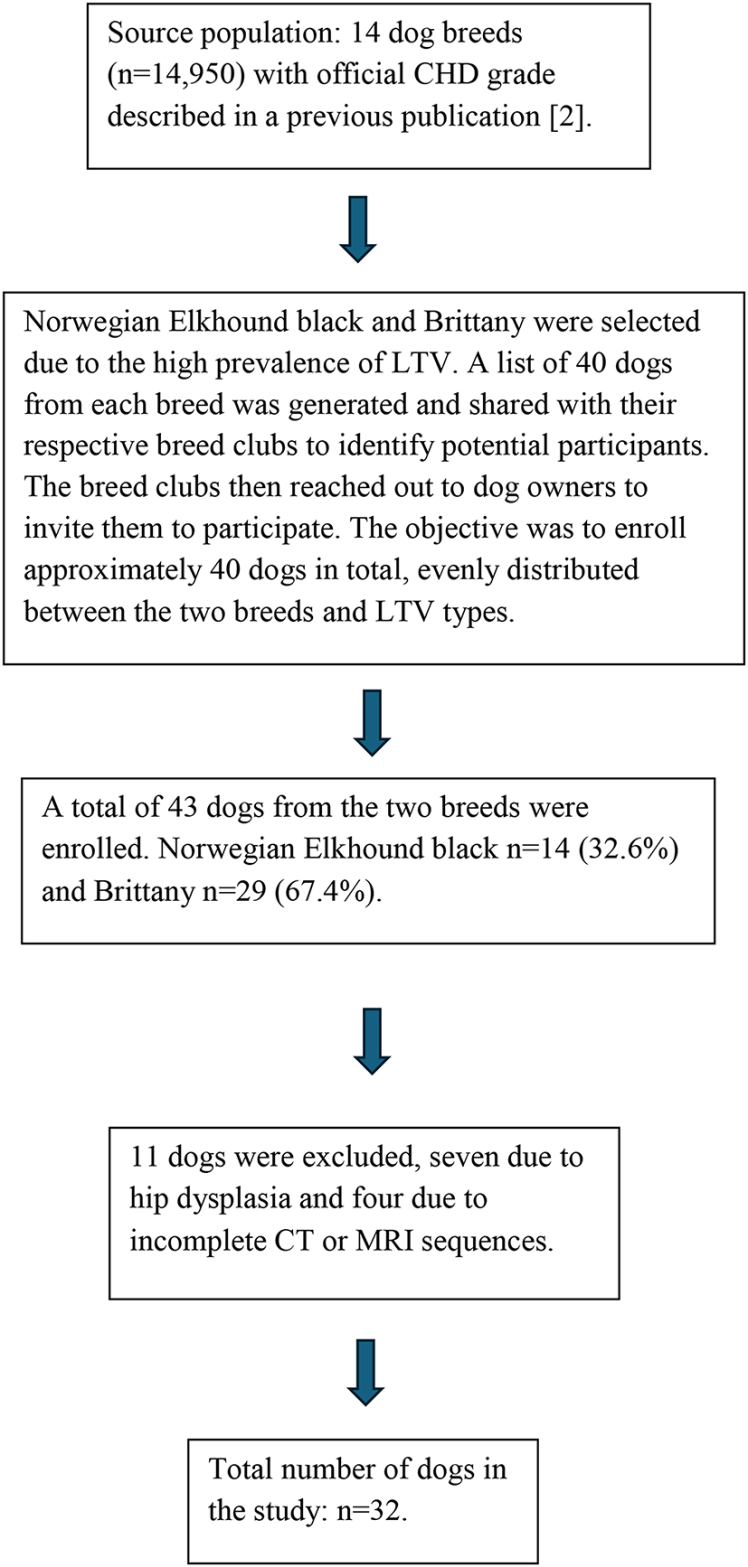
A flow diagram illustrating the process of selecting dogs for the study. The source population consisted of 14 dog breeds. For each dog, we collected the date of birth and an official canine hip dysplasia (CHD) grade from the Norwegian Kennel Club (NKK) [2]. The NKK uses the Fédération Cynologique Internationale (FCI) grading system for CHD, and the evaluation of lumbosacral transitional vertebra (LTV) was based on standard ventrodorsal FCI radiographs. LTV was classified into four types: Type 0 represents normal lumbosacral anatomy; Type 1 is characterised by an independent spinous process of the first sacral vertebra, separated from the medial sacral crest; Type 2 is a symmetrical form where the transverse processes are partially fused with the sacrum or ilium, but the vertebral body remains separate from the sacrum; and Type 3 is asymmetrical, with one side resembling a lumbar vertebra and the other a sacral wing that articulates with the ilium [2, 8]. Our sample included dogs from two selected breeds, based on their home addresses, including dogs with CHD grades A or B (considered free of CHD) and a balanced representation of LTV types across breeds. The target age range was 4 to 8 years, but dogs outside this range were included if their owners were willing to participate. No additional health information was available beyond the details described above

Privately owned dogs from two hunting breeds, Norwegian Elkhound black and Brittany, were included due to their high prevalence of LTV and comparable size [2]. The dogs were selected with the aim of obtaining an even distribution of the two breeds, sex and LTV types 0–3 [2, 8], as well as geographic proximity (no longer than eight hours’ drive to the hospital). All dogs were required to possess an official canine hip dysplasia (CHD) grade of A or B, indicating free from CHD [56] from the Norwegian Kennel Club (NKK). Moreover, we targeted dogs within the age range of four to eight years old, as this is generally the age when dogs are presenting with lower back pain and the diagnosis of DLSS. Dogs outside the preferred age range were not excluded [18, 23, 37]. There was no prior knowledge regarding the dogs’ health (Fig. 1). Dog owners were required to provide written consent to participate. The Norwegian Food Safety Authorities ethically approved this study (Approval ID: 29257).

### Owners’ assessment of pain in their dogs

The owners used the Helsinki Chronic Pain Index (HCPI) to assess pain levels in their dogs [57]. The HCPI comprises 11 questions to quantify owners’ evaluations of their dogs’ pain, with scores ranging from 0 (indicating no pain) to 44 (strong pain). Dogs free from pain usually have an HCPI < 11 [58]. The respective breed clubs sent the HCPI scheme to each owner in advance of their dogs’ clinical evaluation (Additional file 1).

### Clinical examination

All dogs underwent a comprehensive general clinical examination to determine their ASA score and suitability for general anaesthesia [59]. A detailed orthopaedic examination was conducted, and dogs with orthopaedic diseases were excluded [18]. Subsequently, a specific neurological examination targeting DLSS was performed [60], which comprised eight specific tests, a range of minimum score of 0 (indicating maximum severity), and a maximum score of 17 (indicating no abnormality). This examination included the evaluation of posture and gait as well as palpatory manipulation with a focus on the musculature of the hind limbs (quadriceps, hamstrings, tibialis cranialis, gastrocnemius), tail, anal tone and conscious proprioception. Swaying of the back, uncoordinated movements of the hind limbs, spastic gait (reduced flexion), dragging of limbs, and scuffing of toenails are referred to as gait abnormalities.

Pain perception was assessed by the lordosis test, deep palpation of the spine and hyperextending of the tail. Spinal reflexes were evaluated by assessing flexor withdrawals.

Pain severity was classified according to North Carolina State Translational Research in Pain (TRiP); 0: Does not notice manipulation; 1: Orients to the site on manipulation, does not resist or only mild resistance (mild); 2: Orients to the site, slight objection to manipulation (moderate); 3: Withdraws from manipulation, may vocalise, may turn to guard area (significant); 4: Tried to escape from manipulation, or prevent manipulation, may bite or show aggression on manipulation (severe) [61] (Additional file 2).

One clinician (1st author) conducted the examination, unaware of both the owners’ assessment (HCPI) of their dogs’ health and the LTV status.

### Radiological examination

#### Sedation and general anaesthetics

The dogs were pre-medicated with dexmedetomidine at a dosage of 5 µg/kg administered intramuscularly (IM), using Dexdomitor Vet. from Orion Pharma Animal Health (Espoo, Finland), which has a concentration of 0.5 mg/mL, along with methadone at a dosage of 20 µg/kg IM, using Insistor Vet. from VetViva Richter (Wels, Austria), which has a concentration of 10 mg/mL. Anaesthetic induction was achieved by administering intravenous propofol at a 1 mg/kg dosage until effect, utilising PropoVet Multidose from Zoetis Animal Health ApS (Farum, Denmark), which has a 10 mg/mL concentration. This was followed by intubation and maintenance of general anaesthesia with sevoflurane in 100% oxygen, using SevoFlo from Zoetis Animal Health ApS (Farum, Denmark). Monitoring during the procedure included electrocardiography, pulse oximetry, capnography and body temperature measurement. Upon completion of diagnostic imaging, all dogs received intramuscular atipamezole hydrochloride at a concentration of 0.1 mg/mL, available as Antisedan Vet. from Orion Pharma Animal Health (Oslo, Norway), at a concentration of 5 mg/mL. A registered veterinary nurse closely supervised all dogs until they fully recovered from anaesthesia.

#### Radiographic examination

The dogs underwent radiographic examination (Fuji SEDECAL NeoVet Vet 32KW, Madrid, Spain), a neutral lateral radiograph capture of the entire lumbar (L) spine. This included the last thoracic vertebra (Th), lumbosacral junction (LS), sacrum (S) and the cranial parts of the coccygeal vertebra (Ca). This radiograph was used to enumerate the lumbar vertebrae, with the last thoracic vertebra serving as the reference point. A standard ventrodorsal Fédération Cynologique Internationale (FCI) radiograph was also conducted and used for other purposes.

#### Computed tomography (CT)

Scans from L4 to Ca1 were captured in flexion and from the last thoracic vertebra (Th) to Ca1 in extension. The exposure setting used CARE Dose 4D, which adjusts exposure based on the animal’s size relative to reference values; typically, the mAs was 250, with kV set at 110. Scan time was approximately 18 s, depending on the dog’s length, with a rotation time of 1 s and a delay of 3 s. Slice thickness was kept at 0.75 mm, with a pitch of 0.95.

#### *Magnetic resonance imaging (MRI)*

MRI of the lumbar and lumbosacral vertebral column in extended view was conducted using the Siemens Healthineers MAGNETOM Amira 1.5T machine (Erlangen, Germany). During this procedure, the dogs were positioned in dorsal recumbency and supported by sandbags, with their limbs extended in neutral positions and secured with Velcro straps fixed to the MRI table.

For the flexed view, the dogs were placed in right lateral recumbency, with their legs supported by sandbags and secured to the MRI table with Velcro straps.

The imaging protocol comprised various sequences, including transverse plane T2-weighted turbo spin echo (TSE) with repetition time (TR) of 4060 ms and echo time (TE) of 84 ms, coronal (dorsal) plane T2-weighted short tau inversion recovery (STIR) with TR of 3000 ms and TE of 66 ms, sagittal TSE Dixon with TR of 2210 ms and TE of 110 ms, and T2-weighted transverse true fast imaging (TRUFI– 3D) sequence with TR of 8.07 ms and TE of 3.44 ms, each with specific slice thicknesses. Additionally, T1 spin echo in the transverse plane with TR of 614 ms and TE of 12 ms was performed.

All images were acquired and evaluated as DICOM files (Digital Imaging and Communications in Medicine). The field of view for coronal and sagittal views covered from L5 to Ca1, while the transverse plane was the only plane considered during flexion.

#### *Assessment of CT and MRI findings*

All image displays were adjusted, and multiplanar reformatting was used at the veterinary radiologist's discretion (4th author) and reviewed blindly without any knowledge of the clinical findings or information from the owner. MRI and CT studies of the vertebral column, with a focus on the lumbosacral area from L7 to Cd1, were evaluated for the presence of degenerative disc disease, herniation, stenosis of the intervertebral and sacral foramina, degenerative joint disease of the articular processes and sacroiliac joints, spondylosis, telescoping, osteochondrosis, LTV and changes to the nerve roots or cauda equina. Fusion between the sacrum and the Ca1 was subjectively assessed, requiring a minimum closure of 50% of the disc space. Findings were recorded as categorical (present or absent).

### Diagnosis of lower back pain

A diagnosis of lower back pain was based on pain classification (TRiP) during evoked pain when applying LS pressure and inducing the lordosis test. Additionally, identified dysfunctions related to cauda equina were evaluated. The results were combined with supportive advanced imaging findings, having excluded differential diagnoses [16, 17, 20, 23, 62].

### Statistical analysis

Categorical data were reported as frequencies (percentages). Continuous data were reported as mean, median, standard deviation (SD), minimum (min), and maximum (max).

We used Kendall’s Tau-B correlation to determine the relationship between LTV types, DLSS score and the number of lumbar vertebrae.

All P-values less than 0.05 were rendered significant, and all data were analysed using commercial software (jamovi.org, version 2.3.18.0).

## Results

Forty-three dogs were included, of these, 11 dogs were excluded. Seven were excluded because of hip dysplasia, and four because of incomplete CT or MRI sequences.

Among the Norwegian Elkhound blacks, there were seven males (21.9%) and three females (9.4%). Among the Brittany, there were eight males (25.0%) and 14 females (43.8%). The mean age of the dogs was 4.83, SD ± 2.39 years [median: 4.10 (min: 1.40, max: 11.00)], and the mean weight was 14.90, SD ± 2.67 kg [median: 14.50 (min: 11.00, max: 21.10)]. Table 1 provides details related to the age and weight of the two breeds. The overall distribution of the different LTV types was as follows: 9 LTV type 0 (28.1%), 6 LTV type 1 (18.8%), 7 LTV type 2 (21.9%), and 10 LTV type 3 (31.3%). Table 2 presents the distribution of these LTV types between the two dog breeds.

**Table 1:** Signalment data on the two included dog breeds

	Breed	*N*	Mean	Median	SD	Minimum	Maximum
Age (Years)	NES	10	4.45	3.85	1.92	2.00	7.40
	Brittany	22	5.00	4.10	2.60	1.40	11.00
Weight (kg)	NES	10	14.80	14.65	2.31	11.50	19.70
	Brittany	22	14.96	14.35	2.86	11.00	21.10

The table presents signalment data on the two dog breeds used to evaluate the lumbosacral transitional vertebra and its potential association with degenerative lumbosacral stenosis. The sample size comprises 32 dogs: Norwegian Elkhound black (*n* = 10) and Brittany (*n* = 22). NES, Norwegian Elkhound black; N, number of dogs; SD, standard deviation; kg, kilogram

**Table 2:** Distribution of lumbosacral transitional vertebra (LTV) types between the two dog breeds

LTV	NES	Brittany	Tot
0	0 (0.0%)	9 (40.9%)	9 (28.1%)
1	2 (20.0%)	4 (18.2%)	6 (18.8%)
2	3 (30.0%)	4 (18.2%)	7 (21.9%)
3	5 (50.0%)	5 (22.7%)	10 (31.2%)

The table shows the distribution of lumbosacral transitional vertebra (LTV) in the two dog breeds (*n* = 32). LTV types were classified from ventrodorsal radiographs into four types: Type 0 indicates normal lumbosacral anatomy; Type 1 is characterised by an independent spinous process of the first sacral vertebra, separated from the medial sacral crest; Type 2 is symmetrical, with transverse processes partially fused with the sacrum or ilium, but the vertebral body is separated from the sacrum; and Type 3 is asymmetrical, with one side resembling a lumbar vertebra and the other resembling a sacral wing that articulates with the ilium [8]. The table provides the number and percentage of dogs for each LTV type across both breeds and the total sample. NES, Norwegian Elkhound black; Tot, total

According to the owners’ evaluation (HCPI - score), 20 out of 32 dogs (62.5%) had an HCPI score > 1: mean score of 3.40, SD ± 2.26 [median: 2.50 (min: 1.00, max: 9.00)]. Based on this result, where all dogs had a score less than 11, they must be considered pain-free from the owners’ perspective [40].

**Fig. 2:**
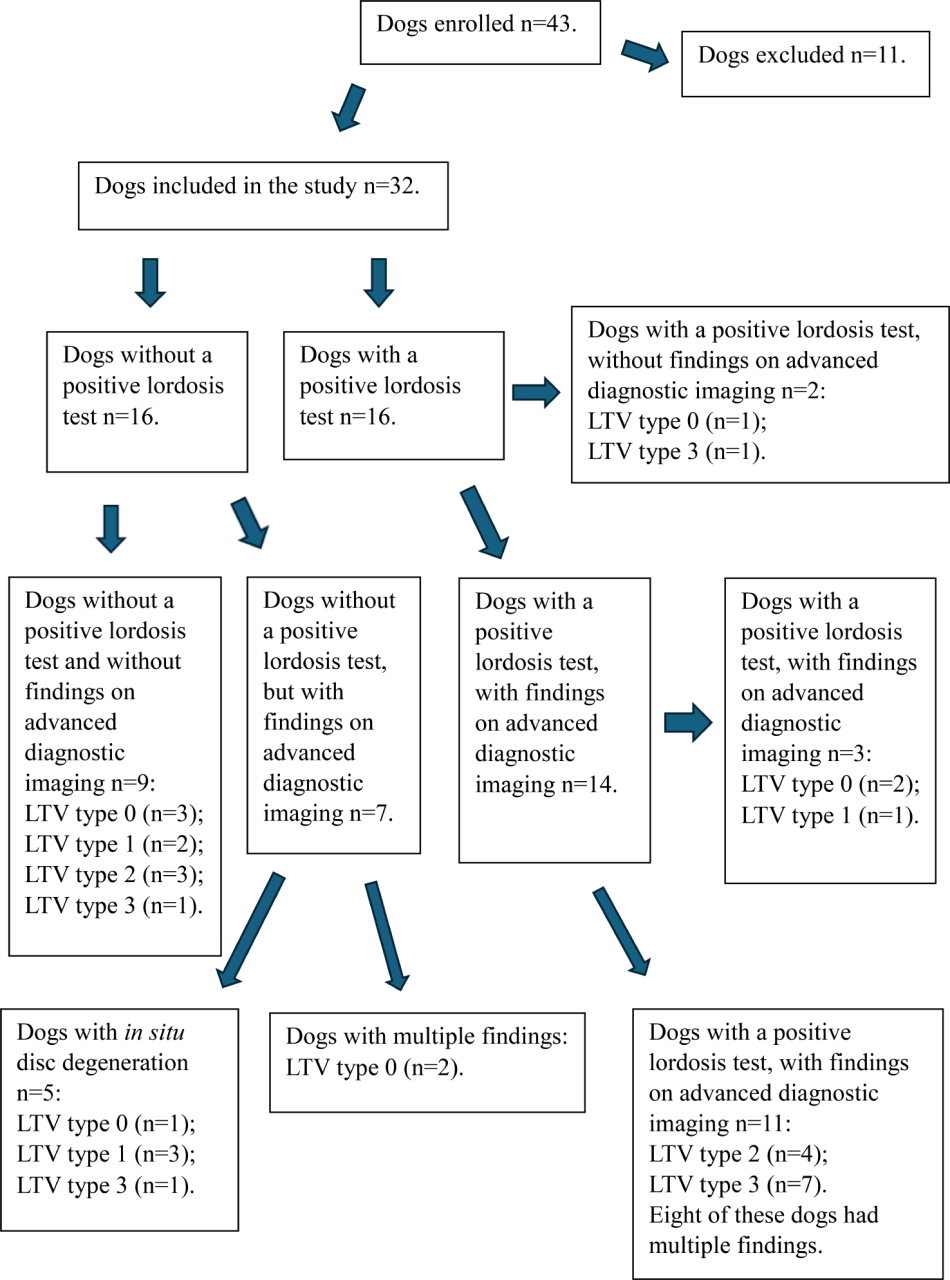
A flow diagram illustrates the dogs’ clinical neurological and advanced diagnostic imaging findings. This flow diagram summarises the findings in dogs enrolled in the study. A total of 43 dogs were enrolled, with 32 included in the study after exclusions. Data is presented for groups based on the findings from the specific neurological examination targeting degenerative lumbosacral stenosis (DLSS) [60]. The findings are reported as binary: presence or absence of a positive lordosis test and findings on advanced diagnostic imaging (CT/MRI). Lumbosacral transitional vertebra (LTV) types (0–3) were classified based on ventrodorsal radiographs. LTV Type 1 is characterised by an independent spinous process of the first sacral vertebra, separated from the medial sacral crest. Type 2 is a symmetrical form in which the transverse processes are partially fused with the sacrum or ilium, but the vertebral body is separated from the sacrum. Type 3 is asymmetrical, with one side typically resembling a normal lumbar vertebra, while the contralateral side resembles the sacral wing, which typically articulates with the ilium. Lastly, LTV Type 0 indicates normal lumbosacral anatomy [8]. All dogs with a positive lordosis test were classified as having mild lumbosacral pain [61]

According to the neurological examination, mild lower back pain was the only recorded remark (positive lordosis test) *n* = 16/32; (50.0%); (DLSS mean score of 15.70, SD ± 0.475 [median:16.00 (min: 15.00, max: 16.00)]). Among these 16 dogs a possible cause for the lower back pain was identified on advanced diagnostic imaging findings in 14/16; (87.5%), where two dogs had LTV type 0 and one had LTV type 1 (Fig. 2).

Among the 16 dogs with no recorded lower back pain on neurological examination (DLSS score of 17), seven (43.8%) showed findings on advanced imaging. Of these, five dogs (71.4%) exhibited in situ disc degeneration, while the remaining two dogs showed multiple findings, including LS disc protrusion, spondylosis, intervertebral foraminal stenosis, and telescoping. Both of these latter dogs had LTV type 0, aged 7 and 9 years. The remaining nine dogs without lower back pain showed no DLSS-related findings on advanced diagnostic imaging (56.3%) (Fig. 2).

**Fig. 3:**
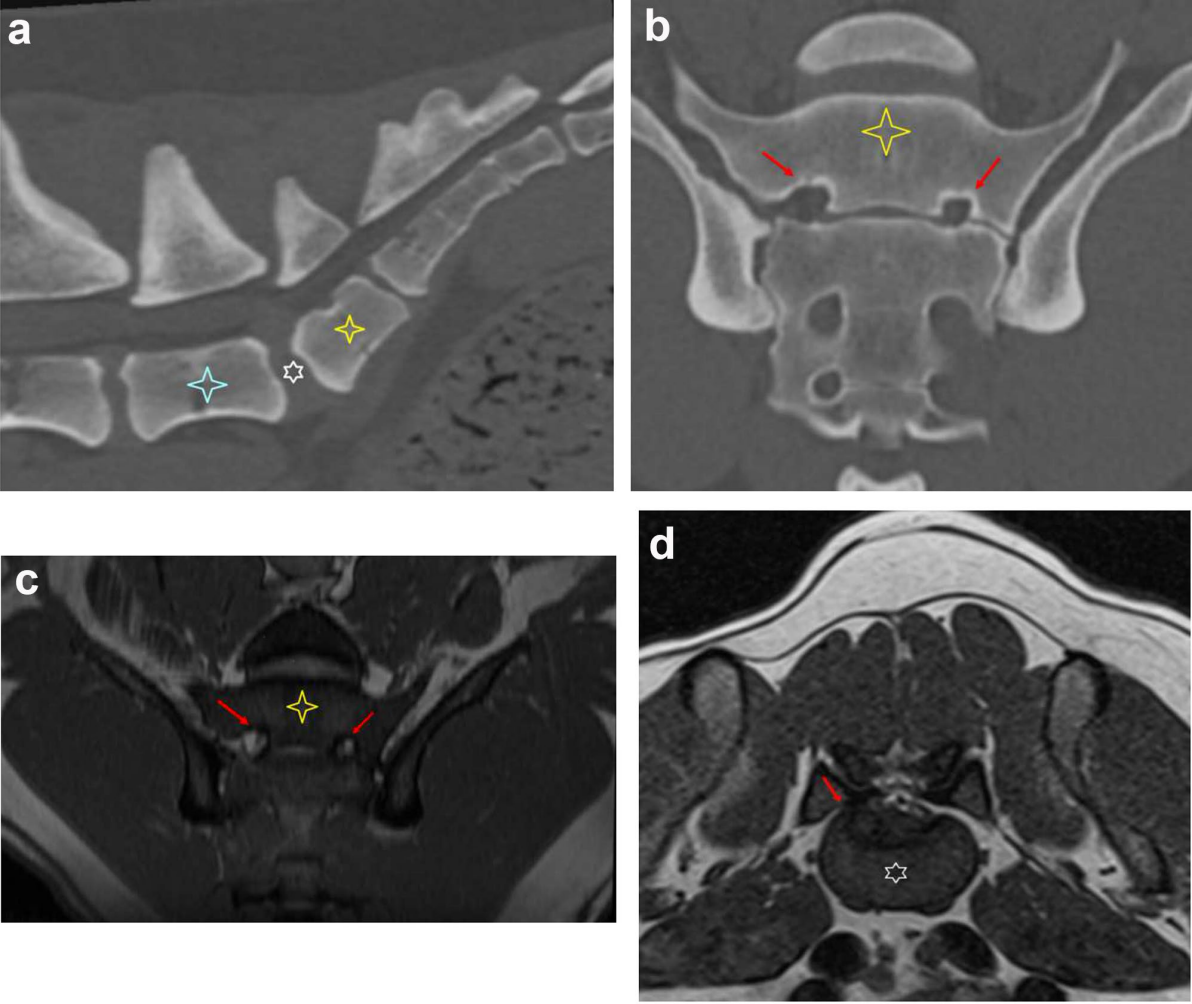
(**a**-**d**) Illustrates a dog with lower back pain and possible source of pain identified on imaging. Sagittal (**a**) and dorsal (**b**) reconstruction, computed tomography (CT) in bone algorithm, and dorsal (**c**) Magnetic resonance imaging (MRI) reconstructed T2 trufi dorsal at the same level, and (**e**) transverse T2 of a Brittany, male, 4.2 years old, with lumbosacral transitional vertebra (LTV) type 2 [8], showing asymmetric sacral foramina (red arrows)– stenosis on the left with partial attenuation of the S1 perineural fat. Protrusion of partly mineralised material into the right ventral aspect of the vertebral canal between the last normal lumbar vertebra and the LTV segment. The asymmetrical appearance of the sacroiliac joints can be appreciated in dorsal images. Blue star indicates the last normal lumbar vertebra. White star indicates intervertebral disc. The yellow star indicates the LTV segment. The red arrow indicates the foramen

**Fig. 4:**
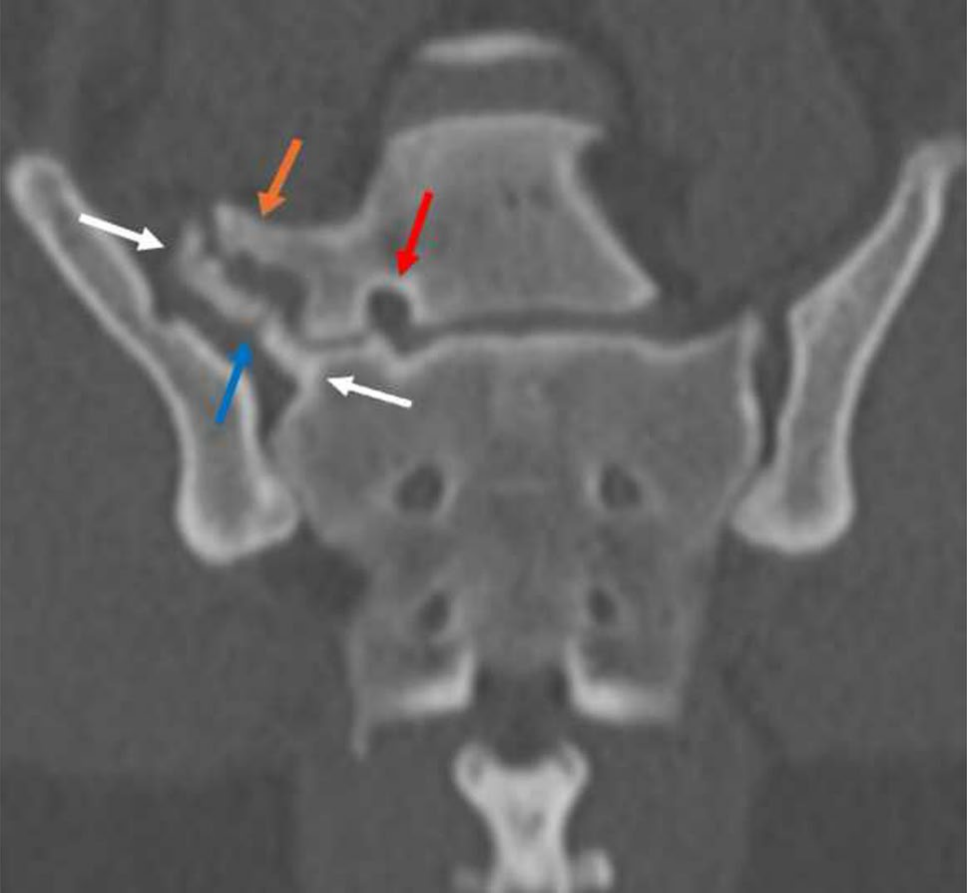
Illustrates a dog with lower back pain and a possible source of pain identified on imaging. Dorsal reconstruction, computed tomography (CT) in bone algorithm, of a Brittany, female, 3.5 years old, flexed position with lumbosacral transitional vertebra (LTV) type 3 [8], showing a right-sided complex connection between the ilium, transverse process of the sacrum (white arrows) and transverse process of the LTV segment (orange arrow), the hypoattenuating thin line in the sacral transverse process is representing a fissure line (blue arrow). The left transverse process of the LTV segment has characteristics of an anatomical normal lumbar transverse process (not shown). Asymmetry of the first sacral foramina, slight sclerosis of the first right sacral foramina (red arrow), in addition to at the base of the right sacral transverse process

In dogs with LTV types 2 and 3 *n* = 17/32; (53.1%), 12 exhibited clinical indications of mild lower back pain, and possible causative pathological changes were observed in 11 dogs via CT and/or MRI (Fig. 2). In most cases, there was more than one pathological finding (Table 3). Figures 3a-d and 4 illustrate some of these findings on advanced diagnostic imaging. Specifically, LTV type 2 *n* = 4/7; (57.1%) and type 3 *n* = 7/10; (70.0%) were associated with lower back pain during neurological examination [τb=-0.393, *n* = 32, *P* < 0.012] compared to LTV type 0 and type 1. These dogs had a mean age of 4.33, SD ± 1.20 years [median: 4.20 (min: 2.50, max: 6.00)].

**Table 3:** Advanced diagnostic imaging findings in dogs with lumbosacral transitional vertebra (LTV) types 2 and 3

Dog No.	Breed	Sex	LTV	No. L	No. Sc	Sacr	DLSS	D.disc	Spon	Prot	IVFS	Sacr FS	Sacroil	Tele	Sum
1	N	M	3	7	3		16	0	0	0	0	1	1	0	2
4	N	M	3	8	2	2	16	0	1	0	0	1	0	0	2
5	N	F	3	8	3		16	0	0	0	0	0	1	0	1
6	B	F	3	7	2	2 + 1	15	1	0	0	0	1	1	0	3
7	B	F	3	7	3		16	0	1	1	0	1	1	0	4
9	B	F	3	8	2	2 + 1	16	1	0	0	0	1	0	0	2
10	B	F	3	8	3		15	0	1	1	0	0	1	1	4
14	B	M	2	8	3		15	1	0	0	0	1	0	0	2
15	B	F	2	6	2	2	16	0	0	1	0	0	0	0	1
16	B	M	2	7	2	2 + 1	16	0	0	1	0	0	1	1	3
17	B	M	2	6	2	2 + 1	15	0	0	1	1	1	1	1	5

The table presents the distribution of lumbosacral transitional vertebra (LTV) in two dog breeds (*n* = 11). LTV was classified from ventrodorsal radiographs into four types: Type 0 indicates normal lumbosacral anatomy; Type 1 is characterised by an independent spinous process of the first sacral vertebra, separated from the medial sacral crest; Type 2 is symmetrical, with transverse processes partially fused with the sacrum or ilium, but the vertebral body remains separate from the sacrum; and Type 3 is asymmetrical, with one side resembling a lumbar vertebra and the other a sacral wing articulating with the ilium [8]. The table also includes data on sex, the number of lumbar vertebrae, and the number of fused sacral bones. The number of lumbar vertebrae was counted on neutral lateral radiographs, starting from the last thoracic vertebra (which is not included in the count). Additionally, it provides each dog’s degenerative lumbosacral stenosis score and findings from advanced diagnostic imaging (CT and MRI). Dog No., Dog number; B, Brittany; N, Norwegian Elkhound black; M, male; F, female; No. L, number of lumbar vertebrae; No. Sc; numbers of fused sacral vertebrae; Sacr, sacral formula 2 + 1 indicating two fused sacral vertebrae partially fused with first coccygeal vertebra; DLSS, degenerative lumbosacral stenosis score [60]; The following parameters are related to findings during advanced diagnostic imaging, where 0 = no finding and 1 = finding; D. disc, degenerated disc; Spon, spondylosis; Prot, disc protrusion; IVFS, intervertebral foramen stenosis; Sacr FS, stenosis of the first sacral foramen; Sacroil, Sacroiliac joint; Tele, telescoping lumbosacral joint; Sum, the total sum of advanced diagnostic imaging findings

**Fig. 5:**
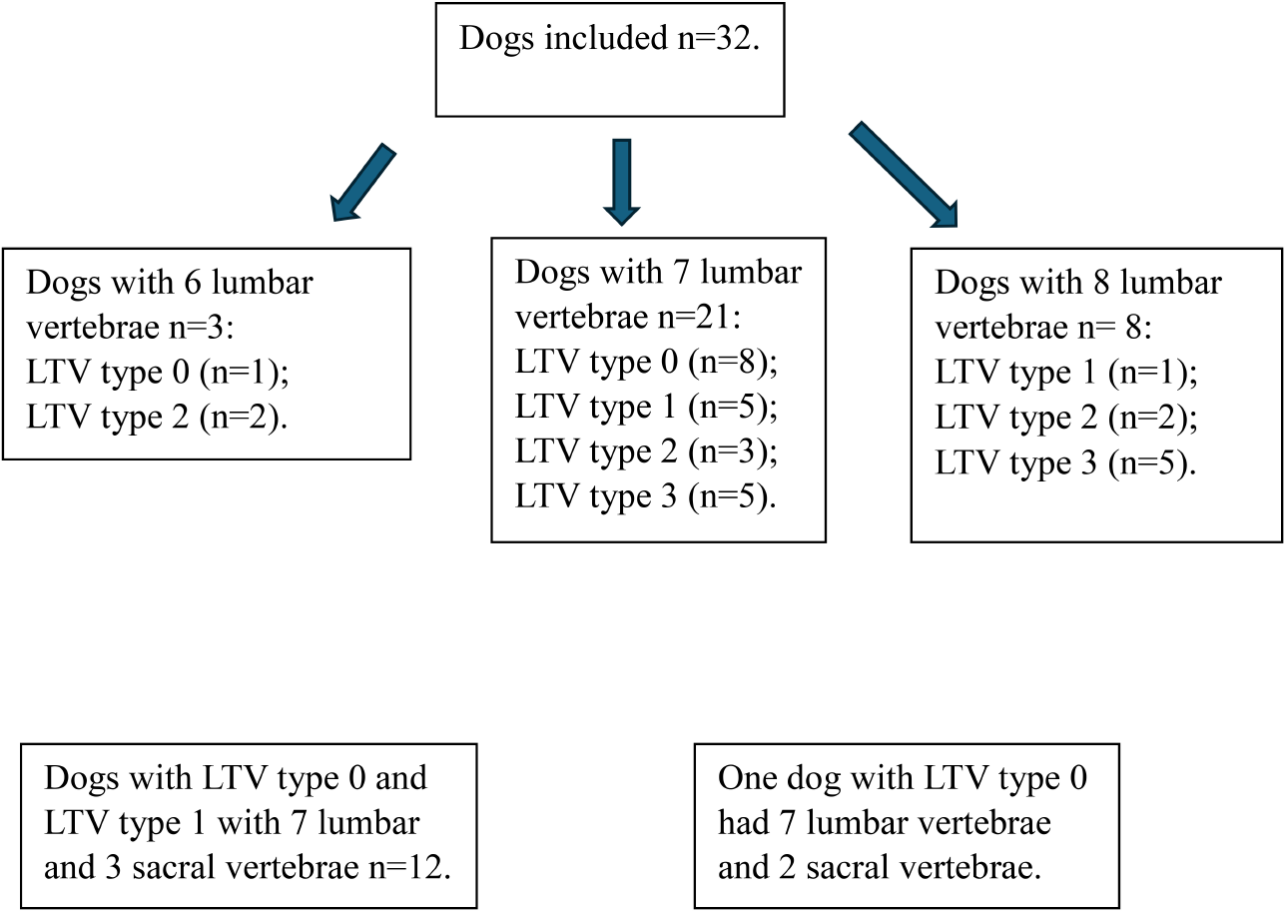
Distribution of lumbar vertebrae numbers and lumbosacral transitional vertebra types in the study population. The included 32 dogs were categorised based on the number of lumbar vertebrae (6, 7, or 8) and their corresponding lumbosacral transitional vertebra (LTV) type (0–3). The number of lumbar vertebrae was counted from the last thoracic vertebra, based on neutral lateral radiographs. LTV types were classified based on ventrodorsal radiographs [2]. LTV Type 1 is characterised by an independent spinous process of the first sacral vertebra, separated from the medial sacral crest. Type 2 is a symmetrical form in which the transverse processes are partially fused with the sacrum or ilium, but the vertebral body is separated from the sacrum. Type 3 is asymmetrical, with one side typically resembling a normal lumbar vertebra, while the contralateral side resembles the sacral wing, which typically articulates with the ilium. Lastly, LTV Type 0 indicates normal lumbosacral anatomy [8]. Notably, 12 dogs with LTV types 0 or 1 exhibited a normal vertebral configuration of seven lumbar and three sacral vertebrae. One dog with LTV type 0 displayed a configuration of seven lumbar and two sacral vertebrae

The number of lumbar vertebrae varied from six to eight, with most dogs having seven lumbar vertebrae [*n* = 21/32; (65.6%)] (Fig. 5). Dogs with LTV 0 and type 1, [*n* = 13/15; (86.7%)], had seven lumbar and three fused sacral vertebrae, while dogs with LTV types 2 and 3 [*n* = 7/17; (41.2%)] had eight lumbar vertebrae (Fig. 5), which was the LTV segment [τb = 0.351, (*n* = 32, *P* = 0.028)] (Table 4). Dogs with two fused sacral vertebrae tended toward fusion with the first coccygeal vertebra, where most of these dogs had seven lumbar vertebrae (Table 5). In six of seven dogs with LTV type 2, the non-fused LTV segment and sacrum were primarily separated by a thin, slit-like disc space that appeared hypointense on T2-weighted MRI images. Only one dog displayed a T2 hyperintense disc in this area.

**Table 4:** The number of lumbar vertebrae, fused sacral vertebrae, and lumbosacral transitional vertebra types (LTV)

Dog No.	No. Lum	No. Sacr	LTV
3	8	3	3
4	8	2	3
5	8	3	3
10	8	3	3
11	8	3	2
14	8	3	2
15	6	2	2
22	8	3	1
26	6	3	0

The table presents each dog with numerical alterations of its lumbar vertebrae formula (*n* = 9). The number of lumbar vertebrae was counted on neutral lateral radiographs, starting from the last thoracic vertebra (which is not included in the count). Additionally, the table provides the numbers of fused sacral vertebrae and the lumbosacral transitional vertebra (LTV) type. LTV was classified from ventrodorsal radiographs into four types: Type 0 indicates normal lumbosacral anatomy; Type 1 is characterised by an independent spinous process of the first sacral vertebra, separated from the medial sacral crest; Type 2 is symmetrical, with transverse processes partially fused with the sacrum or ilium, but the vertebral body remains separate from the sacrum; and Type 3 is asymmetrical, with one side resembling a lumbar vertebra and the other a sacral wing articulating with the ilium [8]. Dog No., dog number; No. Lum, numbers of lumbar vertebrae; No. Sacr, number of fused sacral vertebrae; LTV, lumbosacral transitional vertebra

**Table 5:** Number of lumbar and fused sacral vertebrae across lumbosacral transitional vertebra types (LTV)

Dog No.	No. Lum	No. Sacr	Sacr	LTV
6	7	2	2 + 1	3
9	8	2	2 + 1	3
13	7	2	2 + 1	2
16	7	2	2 + 1	2
17	6	2	2 + 1	2
20	7	3	3 + 1	1
27	7	2	2 + 1	0

The table presents details on each dog’s lumbosacral transitional vertebra (LTV) type based on ventrodorsal radiographs and their lumbar and sacral formulas. Additionally, details pertaining to sacral fusion with the first coccygeal vertebra are provided. The number of lumbar vertebrae was counted from the last thoracic vertebra using neutral lateral radiographs. LTV was classified into four types: Type 0 indicates normal lumbosacral anatomy; Type 1 is characterised by an independent spinous process of the first sacral vertebra, separated from the medial sacral crest; Type 2 is symmetrical, with transverse processes partially fused with the sacrum or ilium, but the vertebral body remains separate from the sacrum; and Type 3 is asymmetrical, with one side resembling a lumbar vertebra and the other a sacral wing articulating with the ilium [8]. Dog No., dog number; No. Lum, numbers of lumbar vertebrae (counted from the last thoracic vertebra, which is not included in the count); No. Sacr, number of fused sacral vertebrae; LTV, lumbosacral transitional vertebrae; Sacr, sacral formula 2 + 1 indicating two fused sacral vertebrae partially fused with the first coccygeal vertebra, 3 + 1 indicates three fused sacral vertebrae partially fused with first coccygeal vertebrae

## Discussion

In this study, we confirmed our hypothesis that clinical signs of lower back pain are associated with LTV types 2 and 3. Based on clinical neurological examination, our results indicate mild pain in the LS area [61–63]. The plausible cause of this pain was identified through advanced diagnostic imaging findings. The earliest and most prevalent clinical indication of DLSS in dogs is evoked pain upon palpation and inducing lordosis of the caudal lumbar spine [16, 18, 20, 64]. While this finding is not exclusive to DLSS, the test is sensitive, showing positive results in 91–100% of DLSS-affected dogs [20, 23, 24]. Based on the findings here, the pain must be characterised as mild [61–63]. This result is not surprising, considering the dogs enrolled for this study.

The occurrence of lower back pain among the included dogs seems high (43.8%). Norwegian Elkhound black and Brittany are not formerly known to be affected with clinical signs of DLSS, which characteristically affects older dogs from larger breeds [20, 23, 24, 37, 65]. LTV has been reported as a risk factor for developing CES in German Sheperd dogs [43, 44], and it has been proposed that LTV might hasten the development of CES in German Sheperd dogs [43]. The high occurrence of lower back pain in this study sample is significantly associated with LTV type 2 and type 3. Among dogs with these LTV types, 64.7% exhibited signs of evoked pain on clinical examination, which could be explained by findings on advanced diagnostic imaging. Given these dogs’ relatively young age and intended use as hunting dogs, this might lead to the progression of clinical signs and may potentially promote early retirement in the future [38, 39].

Advanced diagnostic imaging is considered the “gold standard” for diagnosing DLSS. However, definitive criteria for reliably diagnosing DLSS remain elusive [25–27]. The correlation reported between imaging findings and both clinical and surgical findings has been poor [19, 26, 66]. This issue is highlighted in our observations: two dogs with mild lower back pain showed no advanced diagnostic imaging findings to explain their discomfort, and the two dogs with several pathological findings on advanced diagnostic imaging were without lower back pain. We also encountered cases where dogs without lower back pain exhibited notable remarks on advanced diagnostic imaging, often related to isolated degenerative discs. Degeneration of discs is also considered a normal part of ageing and does not necessarily result in pain [67, 68].

In the present study, dogs with lower back pain are mostly observed with multiple findings in the LS area on CT and MRI [36]. The most frequent pathological findings on CT and MRI in dogs with LTV type 2 and LS pain were related to changes in the disc between the last normal lumbar vertebra and the LTV segment (Fig. 3a, d). This finding is in concordance with previous findings and theorised as a change in the local biomechanics, where a reduced range of motion in the LS junction causes increased stress and range of motion between the last true lumbar vertebra and the LTV, thus hastening the degeneration of this disc [43]. In these cases, we can observe that the slit-like intervertebral disc space between the LTV segment and sacrum was filled with a hypointense material. This finding corresponds to findings in humans [69], indicating a less elastic material than a normal intervertebral disc, which aligns with the theory of adjacent disc disease. It should also be noted that degeneration of the intervertebral disc without compression, so-called discogenic pain, could cause LS pain in dogs [18]. This finding is also reported among younger humans with LTV [70, 71].

In dogs with LTV type 3 and with lower back pain, the most consistent findings on advanced diagnostic imaging were sacroiliac pathology, where the wing-like transverse process of the LTV segment attached to the ilium and/or sacrum, frequently with unilateral stenosis of the ipsilateral first sacral foramen (Fig. 4), followed by intervertebral disc pathology. Disc pathology related to LTV type 3 has been described earlier [33]. Pain and advanced diagnostic imaging findings related to the sacroiliac joint are rarely reported in dogs [45–48] and are not part of the definition of DLSS. The observed diagnostic imaging findings might resemble findings reported in the human literature for Castellvi LTV type 2a. Castellvi type 2 is the most frequent LTV type in humans, showing a high occurrence of lower back pain [53]. The pain is mainly caused by the pseudo-articulation between the LTV segment, ilium, and/or sacrum [72, 73]. However, both “extraforaminal nerve entrapment” and “far-out syndrome” have been reported in humans with this type of LTV [54, 55], but so far not in dogs.

There is inconsistent information regarding the sacroiliac joint radiology findings in dogs with LTV type 3 [33, 44, 48]. Our findings suggest that the sacroiliac joint could be the source of pain (Fig. 4). Considering that sacroiliac pain might mimic several other painful conditions in the LS area, defining a diagnosis of sacroiliac pain remains challenging [74].

**Fig. 6:**
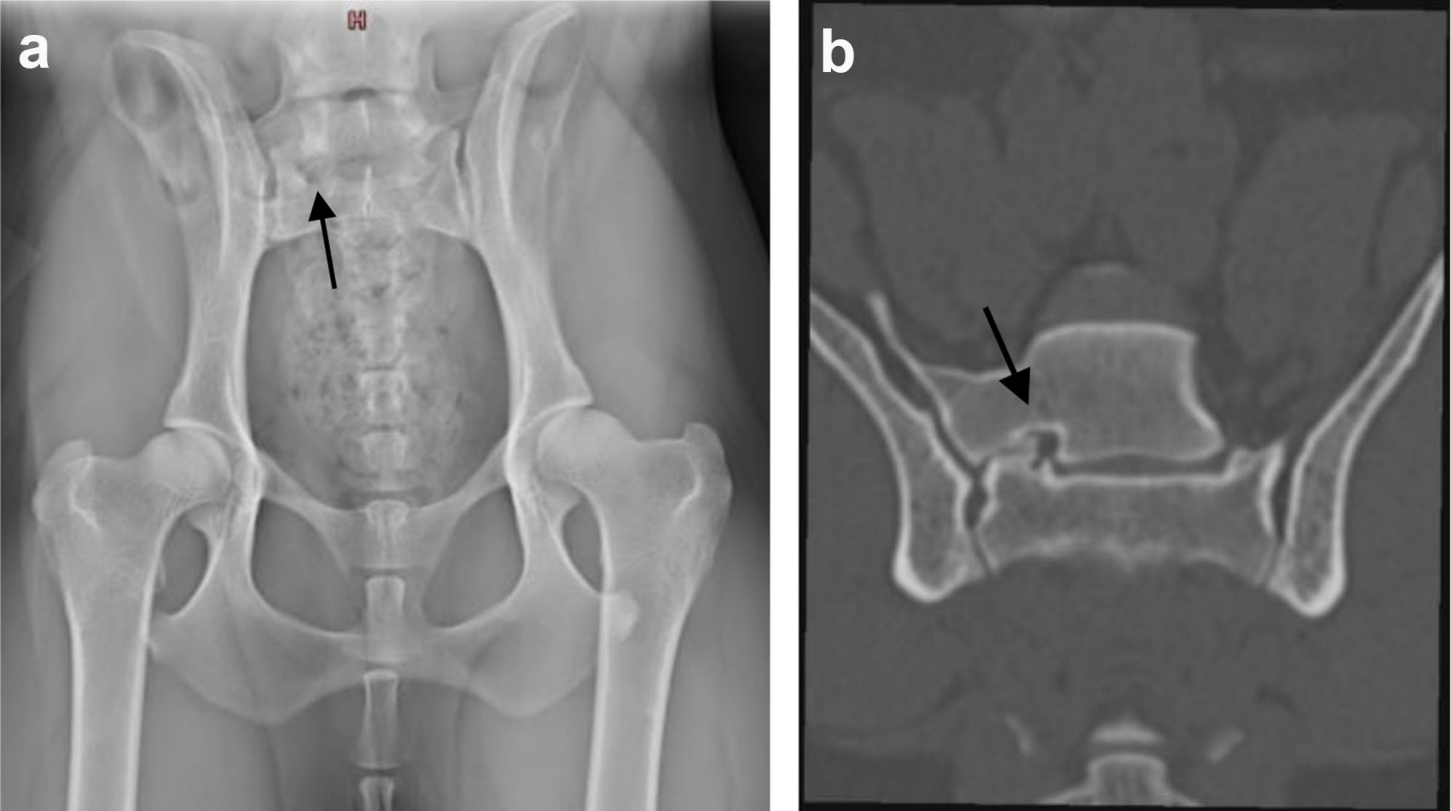
(**a**+**b**) **a**) A ventrodorsal radiograph, **b**) Computed tomography (CT) dorsal bone window. A female Brittany, 3.3 years old, illustrating a lumbosacral transitional vertebra (LTV) type 3 [8] with an articulation of the LTV segment with sacrum on the right side, causing ipsilateral foraminal stenosis of the sacral foramen (black arrow)

Unilateral stenosis of the S1 foramen is also a new finding, predominantly associated with LTV type 3 and visible on plain radiographs (Fig. 6a, b). The ventral ramus of the first sacral nerve supplies fibres to the sciatic nerve [75]. Unilateral nerve impingement is clinically characterised by unilateral pain and progressive lameness, depending on the severity of nerve obstruction, the so-called “nerve root sign” [17].

A phenotypically normal eight lumbar vertebra has been proposed as part of the LTV complex [4]. The eight lumbar vertebra is considered to originate from a non-fused first sacral vertebral body with characteristics similar to the lumbar vertebrae [76]. In dogs with apparently eight lumbar vertebral bodies, the sacrum rarely consists of only two fused sacral bones; it tends to fuse with the first coccygeal bone to form three fused sacral bones [3, 4, 7, 76]. In the present study, the presence of an eight lumbar vertebra was phenotypically abnormal and associated with LTV types 2 and 3. As previously reported and seen here, when the sacrum consists of only two fused sacral bones, it tends to fuse with the first coccygeal bone [7, 76], probably triggered by an intrinsic compensatory mechanism to maintain a sacrum consisting of three fused segments. The fusion of the last sacral bone to the first coccygeal bone has been proposed as part of the LTV complex [76]. In addition, the sacrum occasionally consists of four fused segments [7, 76].

From a clinical point of view, the number of lumbar vertebrae frequently affects the composition of the lumbosacral plexus [77]. The sciatic nerve receives a larger contribution from the first and second sacral nerves in dogs with eight lumbar vertebrae [78, 79]. Based on our findings on advanced diagnostic imaging related to LTV type 3, where unilateral first sacral foramen stenosis was one of the most consistent findings, this may affect the sciatic nerve.

Based on the owners’ evaluation of their dogs’ (the HCPI score), they considered them free from lower back pain. This is in line with a recent study evaluating osteoarthritis in young dogs [80]. Possible causes of inconsistency between the owners’ perception of pain and the diagnosis of DLSS may be that the HCPI score related to pain is not a functional mobility test and does not reflect the impact on the dogs’ daily lives [81]. Additionally, there are established breed differences in pain perception [82]. In human contact sports, athletes develop greater pain tolerance and stamina than those in non-contact sports [83]. Similar traits might be found in athletic hunting dogs, where breeding for hunting instincts and early, consistent training could increase stamina, potentially influencing their pain perception. Differences have also been reported between people who keep dogs primarily for hunting and those who keep them for companionship, regarding pain assessment [84].

It is essential to keep in mind that the pain assessed during DLSS is provoked pain during specific manoeuvres, which is not consistent with being in constant pain, though the threshold is lowered. There has been no thorough description or study of behavioural indicators of pain in young dogs, nor has an owner-questionnaire been developed specifically for this younger population [80]. Previous studies on DLSS are often retrospective and often include surgical candidates. The present study is prospective and includes “healthy” dogs.

Potential limitations of this study include the uncertainty regarding whether the owners were blinded to their dogs’ LTV status, which could affect the HCPI score. The HCPI score has not been validated for DLSS nor officially translated into Norwegian.

The difference between owners’ assessments of pain in their dogs and clinical findings is unsurprising, as the clinical assessment includes tests designed to evoke pain. The fact that owners do not observe any signs of pain in their dogs could also indicate that the lower back pain associated with LTV types 2 and 3 is relatively mild.

A limitation of the study is that lower back pain was identified clinically based on subjective criteria from veterinary examinations. As a result, outcomes may vary if the study is replicated by different investigators. Currently, there is no established “gold standard” for diagnosing or grading lower back pain severity in dogs, highlighting the need for further research to assess the reproducibility of the subjective assessment methods used in this study.

Proton density (PD) MRI and contrast series in CT and MRI could have improved diagnostic imaging accuracy. In this study, we included two different breeds. Therefore, we cannot be certain that the results can be generalised to all dog breeds with LTV type 2 and type 3.

A local nerve block or blocking of the sacroiliac pseudo articulation, which is used in human medicine, could have provided further evidence, but this was not intended when planning for the study [55, 72].

## Conclusions

This is the first prospective study to describe the clinical effects of canine LTV types 2 and 3. In Norwegian Elkhound black and Brittany, the study found significantly higher clinical pain scores for lower back pain compared to LTV types 0 and 1, which were corroborated by changes observed through advanced diagnostic imaging. Thus, dogs with LTV types 2 and 3 with DLSS were younger and lighter than the breeds previously reported. However, the fact that owners did not observe signs of pain may indicate that the pain in these dogs is relatively mild.

## Electronic supplementary material

Below is the link to the electronic supplementary material.

Supplementary Material 1

Supplementary Material 2

## Data Availability

The datasets used and/or analysed during the current study are available from the corresponding author on reasonable request.

## Declarations

### Prior publication

Data have not been published previously.
