# Supplementary material for: Transitional lumbosacral vertebrae in black Norwegian elkhound and Brittany dogs: Clinical findings and its association with degenerative lumbosacral stenosis
Source: Acta Vet Scand. 2025 Feb 12;67:10. doi: 10.1186/s13028-025-00797-7 (PMC11816518; doi:10.1186/s13028-025-00797-7)
Supplement: Supplementary file 1 — Supplementary Material 1 [file 13028_2025_797_MOESM1_ESM.pdf]

NC State Translational Research in Pain (TRiP) Joint Evaluation Scoring SystEm (Canine)

JESSE(Canine)

R Forelimb	Pain (0-4)	Crepitus (0-2)	Effusion (0-2)	Thickening (0-2)	ROM (0-2)
Manus					
Carpus					
Elbow					
Shoulder					
**R Hindlimb**	**Pain (0-4)**	**Crepitus (0-2)**	**Effusion (0-2)**	**Thickening (0-2)**	**ROM (0-2)**
Pes					
Hock					
Stifle					
Hip					
**L Forelimb**	**Pain (0-4)**	**Crepitus (0-2)**	**Effusion (0-2)**	**Thickening (0-2)**	**ROM (0-2)**
Manus					
Carpus					
Elbow					
Shoulder					
**L Hindlimb**	**Pain (0-4)**	**Crepitus (0-2)**	**Effusion (0-2)**	**Thickening (0-2)**	**ROM (0-2)**
Pes					
Hock					
Stifle					
Hip					

Spinal Column	Pain (0-4)
Cervical	
Thoracic	
T-L	
Lumbar	
L-S	

NC STATE ©2019 North Carolina State University, Translational Research in Pain (TRiP)

The descriptors on the following scoring systems are considered a guide and are especially useful for studies and situations where different individuals are making assessments on the same patient over time. Ideally, a single trained individual makes all the assessments on every patient in a given study.

Pain scale based on passive flexion, extension and manipulation

0: Does not notice manipulation
1: Orients to site on manipulation, does not resist or only mild resistance (*mild*)
2: Orients to site, slight objection to manipulation (*moderate*)
3: Withdraws from manipulation, may vocalize, may turn to guard area (*significant*)
4: Tried to escape from manipulation, or prevent manipulation, may bite or show aggression on manipulation (*severe*)

Crepitus, Effusion, Thickening and Range of Motion (ROM) based on passive flexion, extension and manipulation:

Crepitus:
0: none
1: mild, occasional crepitus
2: moderate, crepitus felt always
3: severe, can feel and hear crepitus

Effusion:
0: none
1: mild, small fluid pocket felt only on careful palpation
2: moderate, prominent/obvious on palpation
3: severe, may see visible fluid pocket

Thickening:
0: none, can feel all anatomic structures easily
1: mild, less defined anatomic structures
2: moderate, can still discern the detail of anatomic structures of the joint
3: severe, can no longer feel anatomic structures of the joint

Range of motion:
0: normal
1: mild-moderate decrease
2: severely decreased

NC STATE ©2019 North Carolina State University, Translational Research in Pain (TRiP)
