# Supplementary material for: Transitional lumbosacral vertebrae in black Norwegian elkhound and Brittany dogs: Clinical findings and its association with degenerative lumbosacral stenosis
Source: Acta Vet Scand. 2025 Feb 12;67:10. doi: 10.1186/s13028-025-00797-7 (PMC11816518; doi:10.1186/s13028-025-00797-7)
Supplement: Supplementary file 2 — Supplementary Material 2 [file 13028_2025_797_MOESM2_ESM.docx]

#

# Helsinki Kronisk Smerte Index

Hielm-­‐Bjorkman HK, Rita H, Tulamo R-­‐M. Psychometric testing of the Helsinki chronic pain index by completion of a questionnaire in Finnish by owners of dogs with chronic signs of pain caused by osteoarthritis. Am J Vet Res. 70: 727 – 734, 2009.

(Originalt på finsk, oversatt til norsk fra den engelske utgaven)

*Kryss av i den boksen som beskriver/representerer din hunds atferd best:*

**Vurder din hunds holdning/humør:**

0 1 2 3 4

| Veldig alert | Alert | Verken alert  eller uinteressert | Uinteressert | Veldig  uinteressert/ slapp |
| --- | --- | --- | --- | --- |

**Vurder din hunds interesse for å delta i lek/samspill:**

0 1 2 3 4

| Veldig villig | Villig | Motvillig | Veldig motvillig | Vil ikke delta i lek eller samspill |
| --- | --- | --- | --- | --- |

**Hvor ofte utrykker din hund ubehag/smerte (vasking/slikking/biting på ett spesielt sted, bjeffing, klynking/piping og stønning):**

0 1 2 3 4

| Aldri | Nesten aldri | Av og til | Ofte | Veldig ofte |
| --- | --- | --- | --- | --- |

**Hvor ivrig er hunden til å gå på tur?:**

0 1 2 3 4

| Veldig ivrig | Ivrig | Motvillig | Veldig motvillig | Vil ikke gå i det hele tatt |
| --- | --- | --- | --- | --- |

**Vurder din hunds evne og/eller vilje til å gå opp og ned trapper:**

0 1 2 3 4

| Veldig villig | Villig | Motvillig | Veldig motvillig | Klarer ikke/vil ikke gå i trapper |
| --- | --- | --- | --- | --- |

# Helsinki Kronisk Smerte Index ark 2

**Evaluer hundens evne/vilje til å løpe (trave/galoppere):**

0 1 2 3 4

| Veldig villig | Villig | Motvillig | Veldig motvillig | Løper ikke |
| --- | --- | --- | --- | --- |

**Vurder din hunds evne/vilje til å hoppe (inn i bilen, opp i en stol etc.)**

0 1 2 3 4

| Veldig villig | Villig | Motvillig | Veldig motvillig | Hopper ikke/vil ikke hoppe |
| --- | --- | --- | --- | --- |

**Vurder hvor lett det er for din hund å legge seg ned:**

0 1 2 3 4

| Veldig lett | Lett | Verken lett eller vanskelig | Vanskelig | Veldig vanskelig |
| --- | --- | --- | --- | --- |

**Vurder hvor lett det er for din hund å reise seg fra liggende posisjon:**

0 1 2 3 4

| **Veldig lett** | **Lett** | **Verken lett eller vanskelig** | **Vanskelig** | **Veldig vanskelig** |
| --- | --- | --- | --- | --- |

**Vurder din hunds bevegelighet etter lang hvile:**

0 1 2 3 4

| Veldig god | God | Verken god eller dårlig | Dårlig | Veldig dårlig |
| --- | --- | --- | --- | --- |

**Vurder din hunds bevegelighet under og etter aktivitet (trøtt/sliten, «sleper beina etter seg», subber/sliter ned oversiden av neglene, legger seg ned:**

0 1 2 3 4

| Veldig god | God | Verken god eller dårlig | Dårlig | Veldig dårlig |
| --- | --- | --- | --- | --- |
